# OPTIMUM CIRCADIAN RHYTHM FOR HEALTHY LIFESPAN

**DOI:** 10.1093/geroni/igae098.1909

**Published:** 2024-12-31

**Authors:** Satchidananda Panda

**Affiliations:** Salk Institute for Biological Studies, San Diego, California, United States

## Abstract

To thrive within nature day-night cycle, our genetic makeup is designed to orchestrate the rise and fall of nearly every gene in our genome at specific times. This rhythmic gene expression is vital for maintaining overall health and vitality. In our modern world, circadian rhythms are frequently disrupted, elevating risks of various chronic diseases. An understanding of circadian rhythm principles can lead to lifestyle interventions aimed at preventing, managing, and even reversing chronic diseases. One such pragmatic approach is time-restricted feeding/eating (TRF), a form of intermittent fasting that confines food intake to a consistent 8-10 hour daily window. Laboratory studies have demonstrated that TRF sustains circadian rhythm. TRF can be a preventive or therapeutic measure for several chronic conditions, including obesity, diabetes, liver and kidney diseases, various cancers, and neurodegenerative diseases. Furthermore, TRF, when combined with a healthy diet, can enhance or preserve muscle mass in older animals and extend lifespan. Human studies on TRF are beginning to replicate some, though not all, of the benefits observed in laboratory animals. Beyond food timing, the timing and quality of light exposure also play a crucial role in influencing our sleep and circadian rhythm. Optimal light exposure has been linked to reduced depression, improved mood, enhanced nighttime sleep, and even accelerated recovery in hospitalized patients. In summary, insights from circadian rhythm science are unveiling new lifestyle choices that have the potential to be implemented on a population scale. These interventions aim to sustain both physical and mental health throughout human lifespan.

